# No relation between adenosine triphosphate after manual cleaning and presence of microorganisms on endoscopes after automated high-level disinfection

**DOI:** 10.1055/a-1897-5000

**Published:** 2022-09-14

**Authors:** Judith A. Kwakman, Arjan W. Rauwers, Jolanda G. Buijs, Woutrinus de Groot, Margreet C. Vos, Marco J. Bruno

**Affiliations:** 1 Department of Gastroenterology and Hepatology, Erasmus MC University Medical Center, Rotterdam, the Netherlands; 2 Department of Medical Microbiology and Infectious Diseases, Erasmus MC University Medical Center, Rotterdam, the Netherlands; 3 Qualtity Assurance and Regulatory Affairs Office Medical Devices, Erasmus MC University Medical Center, Rotterdam, the Netherlands

## Abstract

**Background and study aims** Adenosine triphosphate (ATP) tests are increasingly used to detect biological material; however, their reliability to detect bacterial contamination in endoscopes is not proven. We investigated the predictive value of ATP tests after manual cleaning for presence or absence of microorganisms as shown by culture after automated high-level disinfection (HLD) in duodenoscopes and linear echoendoscopes (DLEs).

**Patients and methods** After manual cleaning, ATP tests were performed on swab samples taken from the detachable cap and forceps elevator, and on flush samples of the DLE working channels. These results were compared to the growth of any microorganisms in cultures acquired after automated HLD. ATP tests with > 200 relative light units (RLU) were considered positive. Receiver operator characteristic (ROC) curves were used to compare the RLU levels with microbial presence in cultures.

**Results** In total, 903 procedures were performed involving 26 distinct DLEs. Depending on sample site, 20.8 % (cap) to 63.8 % (channel brush) of the ATP negative samples were accompanied by positive post-HLD cultures. 54.4 % of the cap samples with a positive culture (growth of any kind of microorganism) and 91.8 % of the channel samples with a positive culture had a negative ATP test after manual cleaning. ROC curves per sample site, DLE type and microorganism type all had area under the curves below 0.6.

**Conclusions** In our study, ATP tests performed after manual cleaning could not predict presence or absence of microorganisms after automated HLD as shown by culture. More than half of the positive cultures were preceded by a negative ATP test.

## Introduction

Flexible endoscopes are difficult to decontaminate. High-temperature sterilization is not an option because of heat-sensitive components. Instead, endoscopes are reprocessed, consisting of manual cleaning followed by automated chemical high-level disinfection (HLD). Reprocessing has a very narrow margin of safety and is prone to error which often leads to hazardous situations in which patients are treated with contaminated devices 1 2 . Multiple outbreaks have been reported describing transmission of multidrug resistant bacteria through contaminated endoscopes, causing patient infections and even death 3 . This study focuses on duodenoscopes and linear echoendoscopes (DLEs) since these two endoscope types have a similar complex design and duodenoscopes are most frequently associated with nosocomial infections in gastroenterological endoscopy patients 4 .

Currently, microbiological culturing is considered the “gold standard” to assess the effect or even failure of reprocessing of endoscopes. A downside of this method is that cultures are laborious and results are available only after days or up to a week because of the laboratory process time. Therefore, guidelines recommend to use these cultures as reprocessing quality indicators rather than quarantining endoscopes after every procedure until they are cleared by negative cultures 5 . As a result, transmission of microorganisms to the patient can occur before endoscope contamination is detected. Moreover, microbiological cultures are only representative of the situation at the time of sampling, and their sensitivity to show the presence of bacteria is limited by the sampling and culture methodology. Ideally, presence of microorganisms should be detected within minutes and before each endoscopic procedure.

Point-of-care tests such as the adenosine triphosphate (ATP) test can potentially overcome the long period between culturing and results 6 . Because ATP is present in living cells, it can serve as a substitute measurement for detection of presence of microorganisms in endoscopes 7 . These tests detect ATP using a bioluminescence assay, measuring the emitted light in relative light units (RLUs). A cut-off value > 200 RLUs has been validated by the manufacturer for the most commonly used ATP test (Clean Trace by 3 M), which should distinguish acceptable post-cleaning organic residue levels. However, to measure 1 RLU, at least 10 ^2^ to 10 ^3^ colon forming units (CFU)/mL of a microorganism, without organic soil, need to be present, as was shown in a simulated use study 8 .

The correlation between ATP tests and microbiological cultures has been assessed in multiple studies with conflicting results. A recent review by Olafsdottir et al. found that post-HLD ATP results do not correlate with post-HLD microbiological cultures, but the authors suggested it as a potential quality control measure after manual cleaning 9 . However, the relation between post-cleaning ATP test results and post-HLD cultures has only been clinically investigated in two small (pilot) studies 10 11 . Because ATP tests are being used in a growing number of endoscopy centers 12 and in studies to evaluate manual cleaning efficacy 13 14 , solid scientific data are needed whether this is a reliable and useful measuring method.

In the first part of this study, we investigated whether the contamination of patient-ready DLEs could be reduced by introducing ATP tests to monitor manual cleaning efficacy. The results of that research question have been reported in a separate article by Rauwers et al. 15 and showed no reduction in contamination of patient-ready DLEs. These study data are further investigated in the current study in order to assess whether there is a relationship between ATP level after manual cleaning and presence of viable microorganisms in duodenoscopes and linear echoendoscopes after HLD.

## Patients and methods

The study design was previously described by Rauwers et al 15 . In short, after manual cleaning, ATP samples were acquired and post-HLD microbiological cultures were collected of DLE used in endoscopic procedures in the tertiary care Erasmus Medical Center (Rotterdam, the Netherlands) between July 2017 and October 2018. In April 2018, the endoscopy center was relocated to another building. In the new building, new automated endoscope reprocessors (AER) were installed (WD440 PT, Wassenburg, Dodewaard, The Netherlands), which were connected to reverse osmosis water instead of tap water. In both the old and new AERs, an automated cleaning cycle was followed by an HLD cycle. The study consisted of two phases: 1) the control period during which reprocessing personnel was blinded for ATP test results; and 2) the intervention period in which manual cleaning was repeated if an endoscope tested ATP-positive. In the intervention period, endoscopes underwent the cycle of cleaning and ATP testing up to a maximum of three times before being subjected to automated HLD. The study was approved by the Erasmus MC Medical Ethical Research Committee (MEC-2017–291).

ATP was tested immediately after manual cleaning using the Clean Trace Hygiene Management System for endoscopes (3 M Company, Maplewood, New Jersey, United States). The following sites were sampled: all reachable surfaces of the forceps elevator and cap (if detachable and reusable) were swabbed with a Surface Test, and a 40-mL flush of the biopsy and suction channel collected at the distal end was tested with the Water Test according to the IFU (instructions for use). Following the IFU, a site was considered positive if an ATP test passed the 200 RLU threshold 8 16 .

Directly after automated HLD and prior to drying, cultures were acquired from the forceps elevator and the detachable cap (if present) using a flocked swab (eSwab, COPAN, Brescia, Italy). The biopsy/suction channels were flushed with 20-mL sterile saline solution, half of which was injected through the umbilical suction outlet and the other half through the biopsy port; the samples were collected at the distal end. This volume was chosen because it is the common volume of channel samples according to the Dutch guidelines 17 . After the flush, a brush was pulled through the biopsy/suction channel. This brush was collected in a separate container (eSwab, COPAN, Brescia, Italy). After vortexing, 0.75 mL of the liquid Amies medium (eluent) of the eSwabs used for the forceps elevator, detachable cap and brush samples was poured onto Tryptic Soy Agar. The flush samples were filtered through a 0.22-µm filter and placed on an R2A agar plate. All samples were incubated at 35 °C and reviewed after 4 days for growth, presented in CFU/20 mL per microorganism. Colonies were determined by using Matrix Assisted Laser Desorption Time of Flight Mass Spectrometry (MALDI-TOF MS).

### Statistics

For ATP test results, the range and medians per sample site are given, as these results do not follow a normal distribution. Four different contamination definitions are presented as number and percentage of positive samples per site: 1) gut microorganisms (without oral bacteria); 2) gastrointestinal microorganisms (gut and/or oral bacteria); 3) any growth of any type of microorganism; and 4) growth of ≥ 1CFU/20 mL of gut microorganisms and/or ≥ 20 CFU/20 mL of any other type of microorganism (AM20) as is used in Dutch and European guidelines 5 17 .

We created receiver operator characteristic (ROC) curves for each sample site to investigate whether ATP values could predict presence of microorganisms. ATP results were compared to culture results for the same sample site. Because the working channel was cultured by flush as well as a brush, both of the results were independently analyzed by comparing both to the ATP flush sample of the working channel. To calculate sensitivity and specificity, we compared the ATP results after manual cleaning per sample site with growth of any kind of microorganisms in the accompanying cultures collected post-HLD of the same sample site.

From the procedures performed during the intervention period, we only used the last ATP test results prior to HLD, because the ATP results leading to extra manual cleaning might no longer be related to post-HLD cultures due to the extra manual cleaning. In addition, separate ROC curves were produced to investigate an effect of the different study periods and the four different categories of microbiological presence.

## Results

### ATP results

In total, 903 reprocessing procedures were performed including collection of ATP samples and cultures, involving 26 distinct DLEs. The 3016 collected cultures consisted of 322 detachable cap swabs, 901 forceps elevator swabs, 896 channel flushes, and 897 channel brushes ( Table 1 ). Five channel flush samples and four channel brush samples were lost. The same number of ATP samples were collected from the detachable cap, forceps elevator, and working/suction channel ( Table 2 ). The cap samples were taken from two distinct duodenoscopes, which had a detachable cap. The majority (73.2 %) of the ATP results were below the threshold of 200 RLU. The RLU range for the detachable cap samples was between 0 and 400.120 (median 163), for the elevator samples between 0 and 242.829 (median 113), and for the flush samples between 2 and 19.813 (median 42). The samples of the detachable cap had the most positive ATP test results, with 146 (45.3 %) of the tests exceeding the threshold of 200 RLUs, followed by 306 (34.0 %) of the forceps elevator samples and 116 (12.9 %) of the channel flush samples.

**Table 1:** Presence of gut microorganisms, gastrointestinal microorganisms (gut and/or oral microorganisms), overall microorganisms and AM20 in cultures defined by sample site.

	No. samples	Gut microorganisms		Gastrointestinal microorganisms		Any growth of microorganisms		AM20	
	N	N	%	N	%	N	%	N	%
Detachable cap	322	1	0.3	15	4.7	68	21.1	5	1.6
Forceps elevator	901	16	1.8	49	5.4	304	33.7	37	4.1
Channel flush	896	102	11.4	105	11.7	377	42.1	260	29.0
Channel brush	897	92	10.3	126	14.0	548	61.1	157	17.5
Total	3016	211	7.0	295	9.8	1297	43.0	459	15.2

N; number of positive samples; %, percentage positive samples per sample site; AM20, growth of ≥ 1CFU/20 mL of gut microorganisms and/or ≥ 20CFU/20 mL of any type of microorganism.

**Table 2:** Range of ATP values per sample site.

	No. samples	Lowest value	Highest value	Median	No. of positive ATP samples (> 200 RLU), N(%)
Detachable cap	322	0	400 120	163	146 (45.3 %)
Forceps elevator	901	0	242 829	113	306 (34.0 %)
Channel flush	896	2	19 813	42	116 (12.9 %)
Total	2119	–	–	–	568 (26.8 %)

Of the channel, only a flush sample was acquired for the ATP test. ATP, adenosine triphosphate test; RLU, relative light units.

### Culture results

Flush and brush cultures showed that the suction/biopsy channel was most often contaminated, according to all four categories of microorganisms ( Table 1 ). Growth of any microorganism was shown in 42.1 % (n = 377) of flush cultures and in 61.1 % (n = 548) of brush cultures. The detachable cap was the least often contaminated sample site (21.1 %; n = 68) followed by the forceps elevator (33.7 %; n = 304). Table 1 lists the contamination rates for other microorganism categories per sample site.

### Discrepancy between ATP and culture results

For the majority of cultures that were positive for growth of any kind of microorganism, the corresponding ATP test was negative. This was the case for the detachable cap (54.4 %; 37/68), forceps elevator (72.0 %; 219/304), flush (91.8 %; 346/377) as well as the brush (91.2 %; 500/548). To a lesser extent, cultures that were negative for growth were preceded by a positive ATP test in the detachable cap (45.3 %; 115/254), forceps elevator (37.0 %; 221/597), flush (16.4 %; 85 /519), and brush (19.5 %; 68/349).

Of the detachable cap, 21.0 % (37/176) of the negative ATP samples were accompanied by a positive culture, for the forceps elevator this was the case in 36.8 % (219/595) of the negative ATP samples and 44.4 % (346/780) and 64.0 % (500/781) for the flush and brush samples, respectively. ATP-positive samples were accompanied by negative cultures in 78.8 % (115/146) of the detachable cap samples, 72.2 % (221/306) of the forceps elevator, 73.3 % (85/116) of the flush, and 58.6 % (68/116) of the brush samples.

Of the 903 complete tests performed, 379 (42.0 %) had at least one positive ATP sample, in the other 524 (58.0 %), all sample sites had ATP values below 200 RLU. The majority (346 tests) of these positive tests were acquired in the control period and 81.4 % of the tests in this period had at least one ATP-positive sample. In the intervention period, during which DLEs were manually cleaned up to a maximum of three cycles depending on ATP values, only 6.9 % (33/478) had at least one positive ATP sample. Of the aforementioned combined 379 tests with at least one positive ATP sample after manual cleaning, 125 (33.1 %) had post-HLD cultures without any form of bacterial growth. In 234 (61.7 %) of the cases with at least one positive ATP sample, the cultures were AM20-negative (no growth of gut bacteria and other microorganisms only < 20 CFU). In 429 (81.9 %) of the 524 endoscopes that were ATP-negative on all sample sites, at least one culture was positive for any growth of microorganisms, and 258 (49.2 %) had at least one AM20-positive culture.

### ROC curves

**Fig. 1:**
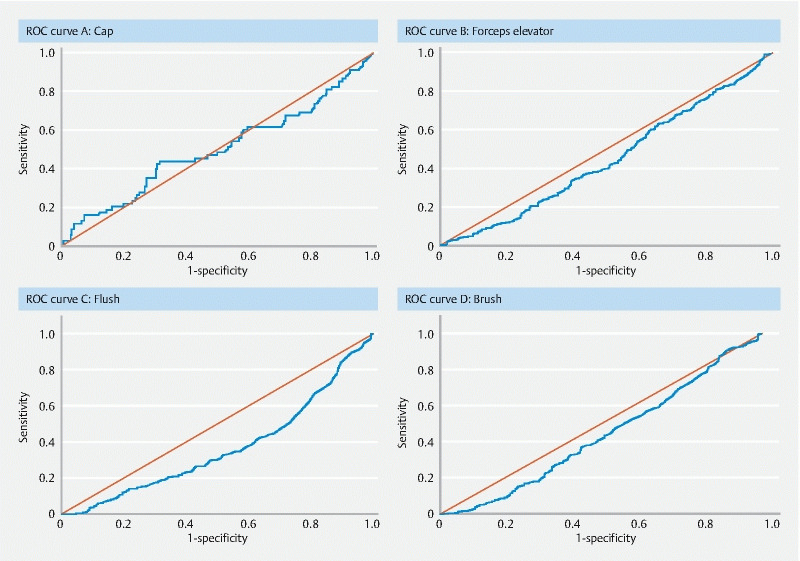
ROC curves correlating ATP outcomes with growth of microorganisms in cultures taken from four sample sites. **a** Detachable cap. AUC 0.498 (0.415–0.582), sensitivity 0.456, specificity 0.547. **b** Forceps. AUC 0.447 (0.408–0.486), sensitivity 0.280, specificity 0.630. **c** Flush. AUC 0.368 (0.331–0.404), sensitivity 0.082, specificity 0.836. **d** Brush. AUC 0.436 (0.397–0.475), sensitivity 0.087, specificity 0.805.

When comparing the post-manual cleaning ATP levels to the post-HLD culture results in ROC curves, we found poor area under the curve (AUC) values ( Fig. 1 ): none of the four main ROC curves had an AUC above 0.5. The AUC of the detachable cap samples was 0.495 (0.411–0.578 95 %CI), for the forceps elevator it was 0.444 (0.405–0.483 95 %CI), for the channel flush 0.362 (0.326–0.399 95 %CI), and for the channel brush 0.431 (0.392–0.470 95 %CI). ROC curves for the three other contamination categories (gut only, gastrointestinal bacteria, and AM20) showed similar outcomes as all accompanying AUCs were below 0.550. Separate ROC curves dividing the two study phases and the two endoscope types all showed AUCs below 0.6.

### Sensitivity and specificity

The sensitivity of the ATP test for samples taken from the detachable cap was 45.6 %, with a specificity of 54.7 %. In samples from the forceps elevator, the sensitivity was 28.0 % and the specificity 63.0 %. The ATP samples from the channel had a very low sensitivity when compared to the flush and brush cultures (8.2 % and 8.7 %, respectively), but the specificity in these samples was high (83.6 % and 80.5 %, respectively).

## Discussion

This prospective study shows that ATP test results after manual cleaning do not predict post-HLD contamination of DLEs as detected by microbiological cultures. Also, no RLU cut-off value was found to be of clinical value since the highest AUC achieved was 0.6 with a low sensitivity and in most sample sites a low specificity as well.

Numerous incidents have been reported in which endoscopes remained contaminated despite being reprocessed exactly according to the IFU and subsequently caused infectious outbreaks among patients 3 . A recent meta-analysis found that approximately 15 % of patient-ready duodenoscopes were contaminated after adequate reprocessing in non-outbreak settings 18 . However, there was much heterogeneity in this meta-analysis, with only 40 % of the included studies reporting the used CFU thresholds to define contamination (> 1 to > 100 CFU) and no differentiation between the types of bacteria found on these duodenoscopes was mentioned. In two nationwide studies in the Netherlands, we also found 15 % of the reprocessed duodenoscopes to contain gastrointestinal and/or oral microorganisms (≥ 1 CFU/20 mL) 1 . As no method has yet been developed to guarantee a zero-contamination rate when using reusable endoscopes, measures to test and control reprocessing efficacy are urgently needed. The gold standard of microbiological culturing is expensive, laborious, and results are known only after multiple days. In contrast, ATP tests are relatively cheap, easy to perform, and give feedback within minutes and, therefore, would be an ideal alternative to culturing. A recent review already demonstrated no correlation between ATP test results and microbial load found in endoscopes with samples collected at the same time after HLD 9 . Several studies have used ATP tests as a surrogate of cultures, with 19 20 21 or without 13 14 comparing the ATP results to culture results collected at the same moment during reprocessing. In this study we used ATP tests as an in-process control and analyzed whether this could predict final presence of microorganisms in cultures acquired after complete reprocessing. The results of our current study are in line with studies by Washburn 22 and Visrodia 10 , in which no correlation was found between post-manual cleaning tests and post-HLD cultures. Visrodia et al. performed a pilot study with a low number of tests in which they also performed ATP tests after manual cleaning and culturing after HLD 10 . They also could not find a relation between ATP and culture outcomes.

We add to this body of evidence that RLU levels post-manual cleaning are not related to the presence of viable microorganisms after reprocessing. Therefore, we believe ATP tests after manual cleaning cannot be used as a substitute for microbiological culturing after HLD to evaluate adequate decontamination of endoscopes. This study shows that if ATP tests were to be used, depending on the cut-off values used to define unacceptable growth of microorganisms, 33.1 % (no growth of any microorganisms) to 61.7 % (AM20 negative) of the ATP-positive endoscopes would undergo unnecessary extra cleaning. Contrarily, 49.2 % (AM20-positive) to 81.9 % (any form of bacterial growth) of the ATP negative endoscopes would be wrongly considered clean enough to continue toward HLD.

The ATP tests in this study were acquired after manual cleaning, whereas the microbiological samples were acquired after automated HLD. The difference between a positive ATP test and a negative culture could be explained by the effectiveness of HLD. Importantly, ATP tests are not designed to specifically identify living microorganisms, but can also reflect presence of other forms of biological debris containing ATP, such as blood or biofilm components, which are not detected by microbiological cultures 9 . An explanation for negative ATP tests in endoscopes found to be contaminated post-HLD might have been presence of microorganisms in such a low concentration and not accompanied by organic soil that they could not be detected by the ATP test 7 8 . Contamination due to the disinfection process itself could also be an explanation. However, in endoscopes that were ATP-negative but with positive cultures, not only environmental but also gut-specific microorganisms were detected. Furthermore, cultures of the final rinse water from the AERs collected during the study did not reveal growth of any microorganisms within these machines.

We found large differences in positive cultures per sample site. The detachable cap had only 21.1 % positive cultures throughout the entire study. The forceps elevator, flush, and brush samples resulted in 33.7 %, 42.1 %, and 61.1 % positive cultures, respectively. This confirms the added value of channel sampling using a friction technique (not only flushing, but also pulling a brush through the channel for physical removal of microorganisms) 23 24 , as the brush samples had a 19 % higher yield than the flush samples collected from the same channels. However, this higher yield was largely due to growth of small numbers of environmental microorganisms (skin and water microorganisms); for the other microorganism categories this higher yield by brushing was not found. Interestingly, the distribution of the ATP results per sample site was in the opposite direction, with the channel testing the least often and the detachable cap the most often ATP-positive. Also, the maximum and median ATP values were markedly lower in the channel samples compared to those of the detachable cap and forceps elevator. This corresponds with the systematic review by Olafsdottir et al. 9 in which higher ATP levels were found after manual cleaning in the elevator samples compared to the channel samples. A possible explanation might be the difference in sampling methods. While the forceps elevator and detachable cap are swabbed to test for presence of ATP, channels are flushed with 40 mL of sterile water. This means that any biological material from the channels is strongly diluted compared to the swab samples. Furthermore, channel flushes used for cultures are filtrated which also contributes to detection of all microorganisms in the channel.

### Limitations

A limitation of this study was the single-center design. In comparison with other ATP studies, we observed high contamination rates and high RLU values 7 13 25 . This might indicate that our center had a problem with accumulation of organic material in our endoscopes prior to the start of the study. Therefore, the setting might not be comparable to other centers with lower contamination rates; however, this is an ideal setting to test the relationship between ATP and culture results. One explanation for the high contamination rate might be that sample collection was performed prior to drying the endoscopes. In previous studies, it was shown that effective drying is an essential step in reducing contamination levels 26 27 . The high ATP levels can partially be explained by ineffective manual cleaning. In the first part of the study, reprocessing staff was blinded for the ATP results, most of the DLEs with at least one ATP-positive sample were derived from that part of the study. In the intervention period, manual cleaning had to be repeated when ATP levels were unacceptable. This might have stimulated better cleaning performance of the staff. However, this effect was not seen in the culturing results post-HLD 15 . We found some very high maximum RLU levels, but these were mostly incidental outliers. Median RLU levels were still below the threshold of 200 RLUs and compared to some other studies, our median RLU levels were comparable or even lower 10 21 . Some of the DLEs with an extremely high ATP result still had negative cultures after HLD, strengthening the conclusion that positive ATP results after manual cleaning cannot predict post-HLD microbial presence. Lowering the threshold to 40 RLU, as is advocated by Ridtitid et al. 19 , would lead to even more positive ATP tests with negative cultures, and thus, to more unnecessary repeated manual cleaning. Finally, it should be noted that the ATP test kit was used outside of the intended use in this study. It is designed for quality assurance of the manual cleaning procedure and not to predict microbial contamination after HLD.

## Conclusions

ATP tests have been advocated to monitor effectiveness of manual cleaning of endoscopes 28 . By selecting endoscopes that would require an extra cleaning cycle, the ATP test should lead to less contaminated DLEs. Although no evidence for this use and effect has been established, use of ATP tests after cleaning has increased in endoscopy centers. This large-scale prospective study shows a low diagnostic accuracy of ATP levels measured on DLEs after manual cleaning compared to presence of viable microorganisms after HLD. Therefore, we conclude that use of ATP tests after manual cleaning does not predict whether endoscopes will be contaminated with viable microorganisms post-HLD.
